# Appraising risk in active surveillance of localized prostate cancer

**DOI:** 10.1111/hex.12912

**Published:** 2019-05-16

**Authors:** Anne Hogden, Kate Churruca, Frances Rapport, David Gillatt

**Affiliations:** ^1^ Faculty of Medicine and Health Sciences, Australian Institute of Health Innovation Macquarie University Sydney New South Wales Australia; ^2^ Tasmanian School of Business and Economics, Australian Institute of Health Service Management University of Tasmania Sydney New South Wales Australia; ^3^ Faculty of Medicine and Health Sciences Macquarie University Sydney New South Wales Australia

**Keywords:** models of care, patient experience, priorities for treatment

## Abstract

**Objectives:**

Men diagnosed with low‐risk prostate cancer are typically eligible for active surveillance of their cancer, involving monitoring for cancer progression and making judgements about the risks of prostate cancer against those of active intervention. Our study examined how risk for prostate cancer is perceived and experienced by patients undergoing active surveillance with their clinicians, how risk is communicated in clinical consultations, and the implications for treatment and care.

**Method:**

Participants were nine patients and three clinicians from a university hospital urology clinic. A staged, qualitative, multi‐method data collection approach was undertaken, comprising: observations of consultations; patient and clinician interviews; and patient surveys. The three data sets were analysed separately using thematic analysis and then integrated to give a comprehensive view of patient and clinician views.

**Results:**

Thirty data points (eight patient surveys; 10 observations of consultations between patients and clinicians; 10 patient interviews; and two clinician interviews) combined to create a detailed picture of how patients perceived and appraised risk, in three themes of “Making sense of risk”, “Talking about risk” and “Responding to risk”.

**Conclusion:**

Effective risk communication needs to be finely tuned and timed to individual patient's priorities and information requirements. A structured information exchange process that identifies patients' priorities, and details key moments in risk assessment, so that complexities of risk are discussed in ways that are meaningful to patients, may benefit patient care. These findings could inform the development of patient‐centric risk assessment procedures and service delivery models in prostate cancer care more broadly.

## 1 BACKGROUND

Prostate cancer (PCa) is the most commonly diagnosed male cancer in North America, Europe,^1^ and Australia^2^ and it is the fourth leading cause of death amongst Australian men, where this study was undertaken.^3^ Although localized PCas (ie those with low histological grade and volume) have a very low risk of metastasizing and are unlikely to be fatal,^4^ untreated, aggressive cancers can metastasize to bone, lymph nodes and other organs, resulting in substantial morbidity and death.^5^

Men with cancer who are assessed as low risk, through prostate‐specific antigen (PSA) testing, MRI and biopsy, that are localized to the prostate, are potentially eligible for active surveillance (AS).^6^ Rather than undertaking active treatments to remove or treat the cancer (such as radiotherapy, prostatectomy and hormonal therapy), AS involves regular monitoring for cancer progression.^7^, ^8^ AS offers patients the choice of avoiding potentially unnecessary active treatments, while enabling timely review and intervention should the cancer progress. Previously termed “watchful waiting”,^9^ which implies a *reactive*, or palliative, approach^7^, ^10^ to changes detected in the prostate (“come back and see me if anything changes”), current AS protocols act as an agreement between clinicians and patients that *proactive* monitoring of prostate health will take place. Appointments for reviewing PCa are often planned in advance, with time frames ranging between 3‐ and 6‐monthly appointments. AS requires the active participation of the patient to ensure that risk is effectively monitored through repeated testing, such as PSA and MRI, and follow‐up urology appointments. The appointment schedule is determined by patients' individual requirements, such as their stage of life, family history, Gleason score (a grading system to identify the aggressiveness of the tumour) and the likelihood of the tumour recurring.

The decision to take part in an AS protocol involves patients, in discussion with clinicians, weighing the risks of regularly monitoring of PCa against those of active treatment, based on the clinician's judgement. The risks of active interventions can include serious side‐effects impacting a patient's bladder and bowel continence, erectile function, fertility and hormone balance,^11^ which then require management from multiple health services. Nevertheless, AS protocols are not without risk of negative effects. Patients may be asked to regularly undergo invasive and discomforting biopsies, ultrasounds, and expensive imaging procedures, such as multi‐parametric MRI scans.^12^, ^13^ Moreover, as it does not involve the eradication of the cancer, AS may be associated with anxiety and uncertainty.^14^, ^15^

Patients' perceptions of risk and their certainty of the effectiveness of treatments for PCa are influenced by their understanding of clinical outcomes,^15^, ^16^ in turn affected by a number of psychosocial factors, such as family support, clinician communication and attitude,^16^ and anxiety and distress.^7^ In this study, we sought to uncover the experiences of men with low‐risk PCa responding to the risks associated with their cancer.. We aimed to identify risk from the perspective of men with localized PCa, who were considering or using an AS protocol to manage their risk of cancer metastasizing, and from the perspectives of their treating clinicians. We asked: (a) “How do patients perceive and experience risk for PCa; and how is risk communicated between clinicians and patients'?” and (b) “What are the implications of patients' perceptions of risk on their treatment and care?”

In this study, we define risk as the probability of a particular clinical event occurring,^17^ such as the risk of PCa metastasizing. Risk is typically expressed to patients in terms of numbers,^18^ to describe probabilities, including percentages of patients who may experience side‐effects from curative treatments, or the likelihood of death at 10 years for those on particular treatments. Even though risk is expressed in terms that are clinical (impacting on a person's physical health) patients can also experience risk in ways that are non‐clinical (impacting on their quality of life) or in terms of psychosocial implications (impacting on their relationship and mental health).^16^ Risk communication in this study builds on our previous work in understanding how risk is discussed with patients, and expressed, in breast cancer.^17^, ^19^

## 2 METHOD

### 2.1 Setting

**Figure 1:**
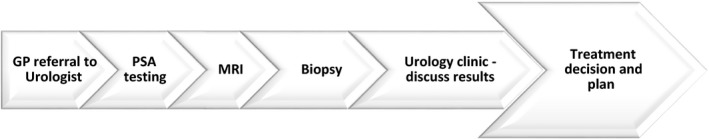
Urology clinic patient pathway for assessment and treatment

The project was conducted in an Australian university hospital urology clinic, between November 2016 and December 2017. The clinic is part of a private practice outpatient service, attached to the university hospital, serving the needs of a diverse patient demographic and offering wide‐ranging clinical services. Ten Urologists see patients in their clinic rooms, and while the senior clinician practises solely at the university hospital clinic, the remaining nine Urologists see patients at multiple clinic sites. General practitioners (GPs) refer patients directly to the Urologists as private practitioners, rather than to the urology clinic. As such, each Urologist's caseload is separate to that of the other Urologists. The Urologists attend a fortnightly multidisciplinary team meeting to discuss their patients collaboratively and to examine patients' cases in detail. The patient flow pattern of the clinic is shown in Figure 1.

As the clinic is a private practice, in this study, the men who used the clinic services were generally from a middle socio‐economic band, living in metropolitan areas and surrounding districts, with a small number travelling up to 2 hours to attend the clinic. The men were typically aged from mid‐40s to 70s and came from predominantly white or south‐east Asian backgrounds. There was a mix of working and retired men, from occupations such as business and trade.

### 2.2 Recruitment

The study was advertised using flyers placed in the Urology clinic waiting room area, to ensure that patients visiting the clinic were aware that the research was taking place. In addition, patients considered suitable for the study (that is, those who were clinically appropriate candidates, who had PCa, were under or considering AS, and showed no psychological sign that involvement would raise anxiety levels or lead to any undue harm) were identified by the two participating Urologists from their patient lists and recruited according to a time frame sampling approach.^20^ Time frame sampling indicates that where a small number of potential participants are involved in clinical consultation over lengthy periods of time, recruitment should encourage all those who consult within that period to be offered the opportunity to participate. People are not conveniently chosen (convenience sampling) rather all patients are approached and all who agree to participate are included. Thus, the time frame is chosen in order to allocate the recruitment period. The approach captures the views and experiences of all consenting eligible participants who fit the appropriate study criteria within a specified window of opportunity, and who are considered suitable (physically, mentally and emotionally) for study inclusion by the clinicians overseeing their care. What is limited is the period of recruitment and not the opportunities for patients to participate, while the sampling technique reduces clinician and researcher coercion and removes researchers from decisions about eligibility. In this study, research team members did not come into contact with patients at the recruitment stage, thus upholding patient anonymity and data confidentiality and while research team members were invited to attend the clinic on the days that patients attended, clinicians discussed the study further with patients before any researcher involvement. If patients were amenable to learning more about the study, one of two researchers (AH and KC), attending the clinic on the day of the patient's visit, made contact to introduce the study to them, offer more information and ask if they still wished to participate. If patients agreed, they signed a written consent form.

### 2.3 Data collection

A multi‐method, staged approach was used to gather data,^21^ comprising: patient surveys; observations of consultations between patients and clinicians; and individual interviews with patients, and with clinicians. Data collection was planned to be consecutive with one stage following the next, so that surveys would precede observations, and be followed by interviews. However, this was not always feasible, as some patients were referred to the study after their consultation appointment, while others were not available to participate in an interview on the same morning as their clinic appointment. Thus, the researchers conducted each stage of the data collection procedure consecutively yet at the convenience of the participants.

The patient survey was purpose designed for the study, based on information gained through discussion with the Urology Clinic team. Survey questions covered demographic and health‐care questions (Appendix S1: Survey questions). Data collection was opportunistic, with surveys completed prior to observations or more commonly at the start of interviews. Moreover, discussion of the survey questions frequently became part of the interview, as participants requested clarification on some of the questions and expanded on their responses.

Observations of consultations took place in each Urologist's consulting room. For most patients, the consultations were the first or second clinic appointment after their biopsy. Sessions were audio‐recorded, and the researcher used a structured observation checklist (Appendix S2: Observation checklist) to note the interaction between clinician and patient. The checklist guided observations of non‐verbal aspects of communication between the patient and clinician, such as body language, and recorded the use of written and visual materials to support medical information.

Participant interviews followed the observation of the consultation. Two interview guides were used—one for patients and one for clinicians (Appendix S3: Interview guide for patients; Appendix S4: Interview guide for clinicians). Interview questions were semi‐structured; that is, the topic of the question was stated, followed by an open‐ended example question that acted as a prompt for further information. This format served to elicit in‐depth responses from participants and allowed the interviewer to ask for more detail if needed. It also avoided any duplication of information if participants had discussed the topic at length while completing the survey.

Patients and clinicians were interviewed in a location of their choice for up to 1 hour. Some chose to be interviewed in the clinic on the day of their consultation appointment, some to be interviewed during the following week from home (face‐to‐face, or by phone), while others chose settings close to the clinic (for example, the hospital café or researcher's office). One patient volunteered to participate over the 12‐month data collection period and to demonstrate an extended patient journey. This patient took part in one survey, three observations of consultations with one Urologist and three interviews.^22^

### 2.4 Data analysis

The analysis process was systematic and iterative, with codes derived from the data and involving contributions from multiple data sources and several members of the team. The three forms of data, in line with a staged approach to data capture,^23^ were first analysed separately. Once complete, the data sets were integrated as one combined data set, to provide a more comprehensive view of patient and clinician perceptions and experiences.

#### 2.4.1 Survey data

Participant data from the demographic surveys were compiled, analysed descriptively and summarized (Table 1).

**Table 1:** Survey data summary (8/9 respondents)

Topic	Category	Subcategory	Result
Health characteristics	Time since diagnosis	Within last few weeks	1
		Within last 3 mo	5
		3‐6 mo	1
		Not diagnosed	1
	Services consulted other than clinic		Radiology × 2
	Health visits for PCa	1‐2	6
		2‐5	2
	Decision for AS or treatment	Decided	6 (4 AS; 1 radiation; 1 surgery)
		Undecided	2
	Awareness of risk classification	Yes	6
		No	2
	Information sources	Doctor in clinic	8
		Doctor in hospital	1
		Nurse/other	1
		Internet	5
		Friends/family	3
		Other	GP; 2nd opinion
	Top ranked priority	Staying physically healthy and living a long life	7/8
		Emotional/mental/social well‐being	1/8
	Lowest ranked priority	Limiting the impact of prostate cancer treatment on your life	5/8
Demographic characteristics	Age range	40‐49	2
		50‐64	2
		65‐79	4
	Sexual orientation	Heterosexual	8/8
	Relationship status	Married	7
		Single	1
	Cultural background	Anglo‐Australian	8/8
	Education level	Uni deg or higher	5
		High school	2

#### 2.4.2 Observation data

Observations of consultations between patients and the two Urologists were undertaken. Eight of the consultations were audio‐recorded and transcribed. Two patients requested that only field notes of their consultations were taken and that the consultations were not audio‐recorded. Observation data were read through by the researcher who had attended the appointment, to identify the relationship between observation notes and interview data and how observations added depth of understanding to interview data, in particular, around risk communication between clinician and patient. Observation findings were assessed thematically, and key themes were merged with themes derived from interviews to form a richer narrative of the consultation that was representative of the interactions between the Urologists and patients.

#### 2.4.3 Interview data

Data were analysed in a stepwise procedure to ensure rigour. First, two researchers independently read through a sample of four patient interview transcripts. Participant statements were coded for meaning in relation to the research question, and study aims and objectives taking account of approaches to transmitting information about risk, communication strategies, and strategies for reducing uncertainty. The researchers then met to discuss the codes that emerged and agreed on an initial thematic coding framework^24^ that could be applied to code the remaining transcripts. Patient and clinician interview transcripts were then uploaded into NVivo 11 (QSR International) to manage the data efficiently and to assist with the understanding of theme development. Once all the transcripts were read as a complete data set, additional codes that were not captured in the initial sample were identified. The coding framework was discussed by the two researchers and amended to complete the preliminary analysis. One researcher grouped the codes to form subthemes as they emerged from the data. For example, the code “clinician's risk representation” formed part of the subtheme “communicating risk to patients” and then rolled up into the overarching theme of “talking about risk”. The themes and subthemes were discussed within the wider research team (comprising health‐care professionals and academic researchers) using tried and tested group work activities^25^ until consensus on key themes emerging from the thematic analysis was reached.

#### 2.4.4 Combined data

Members of the study team reviewed the analysis of each data set and discussed the meaning of the combined findings. Ongoing discussions ensued until a final thematic framework emerged across all study stages. This was supported by the corroboration of observation findings with interview findings, alongside the patients' priorities for their health and well‐being, indicated in the surveys. These combined data sets were reanalysed for agreement or disagreement between data sources. Minor inconsistencies across data sets were discussed and addressed early in the process, leading to a more comprehensive set of findings, for deeper understanding of the data and consideration of further research questions.^26^, ^27^

## 3 RESULTS

In total, 30 sources of data were collected and compiled: eight patient surveys; 10 observations of consultations between patients and clinicians; 10 interviews with patients; and two interviews with clinicians (one Urologist and the specialist Prostate Cancer Nurse, Table 2).

**Table 2:** Data collection summary

Method	Participants		Totals
	Patients	9	
	Clinicians	3	12
Surveys	Patients	8	8
Interviews	Patients	8 initial interviews (7 face‐to‐face; 1 by phone) 2 follow‐up interviews	10
	Clinicians (C1, C2)	2	2
Consultations	C2	6	10
	C3	4	
Total data points			30

Nine men (of 10 approached) consented to take part in the study. Eight men with a diagnosis of localized PCa, who were using or had considered an AS protocol, were enrolled. Of these, one man had previously undergone surgery to remove a high‐risk cancer and was now undertaking AS to monitor any recurrence. The ninth participant did not have a PCa diagnosis, but had a strong family history of prostate cancer, who regularly undertook screening tests for onset of the disease that was akin to the AS protocol used in the clinic (see demographic data below for more detail on the patient cohort).

Clinician participants were two male Urologists (four Urologists were approached) and a male specialist Prostate Cancer Nurse. The three clinicians participated in different data collection activities. The two Urologists participated in observations of consultations, but no consultations with the specialist Prostate Cancer Nurse were observed. One Urologist and the specialist Prostate Cancer Nurse took part in interviews.

Of the nine patients considered suitable for an AS protocol, seven remained on AS, one opted for surgery and another for radiotherapy. Of the consultations, six were with one Urologist, and four were with the second Urologist. Patient consultations with the specialist Prostate Cancer Nurse were not observed.

Combined data generated a detailed picture of the way patients and clinicians perceived, discussed and responded to risk in this care setting. Disagreement between data findings was minor and therefore is not presented; for example, the number of patients reporting using friends as an information source differed slightly between survey and interview data. Three themes integrated the findings to represent patients' appraisal of risk. These were “Making sense of risk”, “Talking about risk” and “Responding to risk”. Quotes illustrating each theme are placed in tables and referenced to the related text. Participants are identified as either patient (P) or clinician (C).

### 3.1 Making sense of risk

Patients reported making sense of the risks posed to them through understanding the meaning of their diagnosis, how it impacted on their personal priorities for their lives, and the information they accessed to come to terms with their situation. Most patients were recently diagnosed, and their understanding of the risks posed to them by PCa and participating in an AS protocol was new. Patients did not distinguish between clinical risk, non‐clinical risk and psychological risk, but in how PCa impacted on their length and quality of life, and whether the effect was of a physical, mental or emotional nature. Participants recalled their reactions to receiving the diagnosis of localized PCa, with many reporting feelings of shock at the diagnosis. Some struggled to understand what it meant to their life, and how they should respond. Others drew on long‐held philosophies and personal beliefs to deal with their situation (Table 3, quote 1).

**Table 3:** Making sense of risk: Participant quotes

Number	Quote
1	“I've got to attack it, but at no time during this was I anxious. To me, it was uncertain, but the uncertainty didn't turn into anxiety. I was just going to do it as an engineer, I mean, it could have got worse, but I just didn't know which way I was going to go”. P6
2	“Well, there's … shortened life in terms of, you're dead …shortened quality of life, excluding sex. That means, are you having a problem pissing, are you hindered [from doing] other things you can't do?... And thirdly, which, I've sort of held off separately, the enjoyment of sex.” P5
3	“I don't want the prostate ripped out because then that's goodbye to any chances of family”. P1
4	“I personally like to know what the real sort of risks are, and you look for some sort of comfort in the statistics in that they might say only 10% of people will progress from this stage, or whatever it might be, I guess that's what I was sort of looking for… at that point, you're just looking for some sort of reassurance, and some sort of higher level of—some way of minimising the worry”. P2
5	“With the websites, not being a medical professional, having that much knowledge around these things, I think it's dangerous to actually read them and infer any conclusions from it, because there can be such subtle differences that you just don't appreciate, don't know about, that can totally change it. For example, I was thinking at one stage, well, it says 20% of people might have cancerous cells by the time they're 50 … It's just a bit more information to understand, which is good and bad. You find good things, but you find bad things when you read that. That was my initial experience”. P2
6	“You've got to be a bit careful with the Internet ‘cause there's a lot of loonies out there that put stuff on”. P4
7	“At my age so many of my friends are now having it, so—and I've got another mate who didn't take any notice of it at all and he's now on major chemo”. P4
8	“I've known blokes in their 70's who died very quickly, once it's sort of in you, it metastasises; but mine hasn't”. P7
9	“I got a lot of information from my father who had done quite an extensive research into it, but I've also done my own research on the internet … they were generally all saying the same thing. I went to medical websites but then also patient websites just to get their feedback and what they thought from the different options. And my father did quite a lot of research into that and was using his knowledge as well”. P8

Risk was frequently perceived and expressed through the lens of patients' personal priorities. Participants' broader priorities, when selected from the survey categories, were frequently held in common. For example, “Staying physically healthy and living a long life” was most frequently identified as a top priority, while the lowest ranked was “Limiting the impact of prostate cancer treatment on your life”. Nevertheless, patients' perceptions of their own risks from PCa were more varied and nuanced when described at interview. Some defined their fears and risks in relation to their personal priorities, in concrete terms of how it might affect their quality of life, length of life and plans for their future (Table 3, quotes 2 and 3). Others made sense of their risks through statistical information, applied to their goals for living. They found reassurance in publicly available information that helped them understand what it meant to be in a low‐risk category (Table 3, quote 4).

Participants identified a range of information sources they had used to understand their condition, their risks and what the future might hold, in addition to what was provided by their clinicians. These sources included a combination of Internet‐based information and family and friends' experiences. Patients wanted Internet‐based information, but expressed difficulty in identifying information that related to them and their own risks. The Internet offered a broad range of information, but patients did not have the knowledge to read it selectively. Some reported feeling alarmed by information about advanced cancers and not knowing if this was their future (Table 3, quotes 5 and 6).

Several participants reported drawing on friends who had experienced PCa as a source of support and information, but also as a warning. Learning from the experiences of friends influenced patients' appraisals of risks and complexities associated with the disease, and with treatment options (Table 3, quotes 7 and 8).

One patient with a family history of PCa (father and grandfather) drew on their collective experiences to motivate vigilance of his prostate health and to keep himself up to date with detection and treatment procedures (Table 3, quote 9). Patients also drew on information given to them by their clinicians during consultations. This is outlined in the following section, describing how risk was discussed in clinical consultation.

### 3.2 Talking about risk

During consultations, clinicians used a patient‐centred approach to identify the most appropriate prostate cancer intervention for each patient. This approach was evident in the use of shared decision‐making principles (such as discussion of evidence‐informed strengths and weaknesses of the treatment options, with reference to the patient's values and preferences). The approach was put into practice to support patients to reach their decision from the way information was presented to the patient, and the interaction between patient and clinician, both verbal and non‐verbal. Both Urologists adopted a similar approach to patient care, and the body language observed between patients and Urologists indicated that patients seemed relaxed and comfortable throughout their consultation and in discussing intimate details about their health and health‐care needs.

Interactions between patient and Urologist were generally led by the patient, either by presenting new information to the Urologist, checking that test results had been received, or requesting information about specific issues. Urologists responded by providing evidence‐based information from academic publications about the issue under discussion, for example, recommendations for diet or exercise. Information from the Urologists was delivered verbally, supported on occasion by use of anatomical models to clarify information about the prostate. Many patients brought notes as reminders of the concerns they wished to discuss, and these were raised with the Urologist as part of information sharing. Information about risk, in terms of survival rates, was presented verbally by the Urologist if the patient queried their lifetime risk or chance of survival.

During consultations, clinicians communicated risk information in several ways. Firstly, Urologists went through the assessment procedures used to diagnose and classify the cancer (PSA, MRI, biopsy), and which of those would be used to monitor change in the cancer (PSA, MRI). Secondly, clinicians described the tumour using Gleason scores, interpreted in lay terms for the patient. Thirdly, clinicians responded to patients' concerns about their risks from PCa by providing reassurance and addressing patients' specific issues as they arose in the discussion (Table 4, quotes 10 and 11).

**Table 4:** Talking about risk: Participant quotes

Number	Quote
10	“They probably think they've been given a diagnosis of ‘you're going to die', so giving reassurance first of all—firstly, [there's] no immediate danger, and secondly you need to actually get the right treatment to get a long and healthy life hopefully”. C2
11	“Sometimes it's purely sitting down with the patient and just saying, ‘What are you worried about?' You give your standard spiel, but then you go, ‘Well, what are you worried about? What's the big bugs for you?' Especially it's a great thing if they've got the family [with them] because they can have different worries, ‘Well, what are you worrying about?' If the kids are there, ‘What are you worried about?'” C1
12	“Even on a low‐grade cancer like yours, for example, if you leave them alone, 95% are going to be all right”. C2
13	“If you haven't got a prostate you can't be fertile; it's impossible. And usually as you get older you sort of discount that. But I've known a few people in their 50s, 60s, who still think about having children, so you need to take that into consideration”. C2
14	“Dr [Urologist] was very good, he was very supportive and there was another guy, I can't remember his name, he was a Nurse in that area. Nice guy, really nice guy. Between the two of them, they made sure that I was happy with what was going on, where I wanted to go, and they gave me all the scenarios of what I should do. I'm very grateful to both of them, they were both really good”. P4
15	“If the risks are, from what I can see, low, and it's not caused by my lifestyle, therefore there's nothing to change. I'll just carry on as normal”. P1
16	“He's got a guy called [Nurse name] who is a very helpful guy. So, if I had a change I'd probably give [Nurse name] a call and say, hey I've got this change, should I come in and see the guy [Urologist]?” P3
17	“I'm in the hands of the medical expertise, and if they said, ‘Oh, we've got to slam you in hospital tomorrow and rip your prostate out, otherwise you're going to die,' of course I'd do it. But given the fact that the advice I've received is Active Surveillance, it tells me that the risks are low, therefore I'm not doing terribly much about it”. P1

Clinicians communicated risk to patients using statistical probability information, applied to each patient in terms of their age, health status and life goals (Table 4, quotes 12 and 13). Patients requesting reassurance about lifestyle behaviours, such as their diet and exercise, were given advice based on emerging research information delivered verbally by the clinician.

Once the patient's concerns had been addressed, treatment options appropriate to the patient's needs, including information about an AS protocol, were presented by the Urologist. The benefits and disadvantages of each potential treatment were made clear to the patient. A follow‐up plan was then negotiated, guided by the needs of each individual patient, and the recommendations for PCa care were offered. The consultation concluded with the Urologist's report to the GP being dictated while the patient was present. This ensured that patients were aware of the information being transmitted to their GP. While not observed, clinicians reported that health literature on PCa was made available during consultations with the specialist Prostate Cancer Nurse, alongside information about prostate cancer support services.

Patients expressed satisfaction in the care they received through the clinic, and trusted their clinicians' judgement (Table 4, quotes 14 and 15). Patients and Urologists valued the availability of a specialist Prostate Cancer Nurse for booked appointments and phone consultations. The Nurse's role was viewed as interpreting information and confirming advice after patients' medical consultation. The Nurse was available to be contacted throughout the week, if patients had any concerns they had not raised during appointments. Perhaps related to this availability, patients reported feeling more confident to contact the Nurse than the Urologist (Table 4, quote 16).

However, as a new service, not all patients were aware that the Prostate Cancer Nurse was available to them. Those patients unaware were informed of the service by the researcher. Once patients understood their risks, and how they could be proactively monitored through an AS protocol, they expressed confidence in how their prostate health was being managed by the clinic team (Table 4, quote 17).

### 3.3 Responding to risk

As patients gained understanding of the risks they faced, they sought ways to take control over their health. This frequently took the form of self‐management approaches to minimize risks, reduce anxiety and uncertainty, and determine their way forward (Table 5, quotes 18 and 19). A common approach was in seeking reassurance about their personal risk from PCa, to gain certainty about their treatment decisions. When patients had made their decision, many wanted the Urologist to confirm whether he thought they were making the right decision, by asking the Urologist what he would choose under the same circumstances (Table 5, quote 20). Moreover, the Urologists frequently framed their recommendations as what they themselves would do if they were the patient (Table 5, quote 21). One patient surmised the relief expressed by many, when they were able to fully appraise their risks, and move on to making management decisions for PCa (Table 5, quote 22).

**Table 5:** Responding to risk: Participant quotes

Number	Quote
18	“I had to sort of pick myself up and say; ‘what am I trying to do here?'... So then I had two opinions … So I will now take management back … I can make sure it's monitored which I'm doing. I can yell out if there's any change in any of the activity”. P3
19	“I can understand the different roads to be taken and the risks associated with each one. I plotted in my mind a map that works, and I think is logical for everyone's point of view”. P4
20	“P6: If it was your prostate, what would you do? C3: I'd have radiotherapy. I think it's going to get a better shot at you, basically, with it. You're also 73. The side effects of radiotherapy are less likely to affect you, versus a 53 or 63‐year‐old”.
21	“C2: If it were me, I'd probably watch the—I would watch the PSA, go for a MRI and then, you have a repeat biopsy as something on, you know, the persistent rise and the PSA and unless there's something on the MRI that wasn't there before … P5: Okay. So I'll just do the PSA in three months and then, PSA and see you in six”.
22	“I was just so relieved to hear that there was nothing else found and that he was recommending the [Active Surveillance] that I thought, well, regardless of if it's a 20% chance of it progressing or a 60% chance of it progressing, what I need to do now is, is watch and wait and not worry about it, because I don't want to go through my life knowing that you're on a bit of a ticking time bomb”. P2

## 4 DISCUSSION

The findings from this study add to previous research in PCa care that has investigated patient decision making in PCa diagnosis and treatment,^16^ such as men's understanding of benefits and harms associated with PSA testing^28^; and their decision‐making and treatment preferences.^29^, ^30^ However, in this study, we have concentrated on risk, through qualitative interviews, observations and surveys. The evidence in our study suggested that effective risk communication in PCa may benefit from fine tuning to individual patient's priorities and information needs. Based on evidence from observation of consultations and patient and clinician interviews, we propose a structured approach to information provision that emphasizes that informed decision making about interventions for PCa is a multilayered *exchange* of information.^31^ In this way, our findings build on previous recommendations for communicating risk in cancer, identifying helpful modes^32^, ^33^ and structures^31^, ^34^ for risk communication.

Clinicians in this study demonstrated that they balanced clinical judgement with evidence‐based information, to deliver a patient‐centred approach when discussing risk in PCa, in response to the perceived needs of patients. Discussions of risk centred on understanding patients' priorities and preferences, to help them decide whether to choose or remain on AS or pursue active intervention.^35^ Reflecting the preference‐sensitive nature of this decision, clinicians gave assurances without explicitly directing patients towards an option and respected patients' choices. Furthermore, access to a specialist Prostate Cancer Nurse within the clinic contributed to patients' understanding of their cancer and the risks it posed to their survival,^36^, ^37^ quality of life and psychological well‐being, as well as offering them reassurance that their PCa was being actively monitored using a protocol that reflected their priorities. Clinicians outlined individuals' assessment results, and an estimation of their clinical risk, including rates of survival after 10 years, and addressed the pressing psychosocial concerns that patients raised, such as maintaining sexual activity.

Timing was central to patients receiving the information they required to reduce their uncertainty and make treatment decisions.^14^ For example, by their second consultation, after their biopsy, patients had received the information they needed about their tumour and the risks it posed to them. But it was the time preceding this, when patients did not understand their own risks and the implications of risk for their future health and well‐being, that created anxiety and uncertainty. Information gaps were mostly resolved after two clinic appointments, when there had been time to raise concerns with a Urologist and specialist Prostate Cancer Nurse. However, at the beginning of the patient journey, much was unknown—such as what risk meant, which risks personally related to an individual patient, and the full extent of the clinic service on offer. It may be that earlier risk information provided between first and second appointments following diagnosis would be an optimal solution to information provision when patients most need it. This finding complements that of Taylor et al^14^ who found that discussion of AS almost immediately after the patient had been diagnosed was important to appropriate uptake of AS, reducing inappropriate and ill‐informed choices for active treatment.

Patients used information‐seeking strategies to alleviate their anxiety. Although they wanted certainty, participants did not explicitly ask clinicians which option they should take. Instead, they sought assurance from clinicians by asking if the option they had chosen matched what the clinician would have chosen for themselves, had they been in the same situation. This finding adds depth to the views of Hoffman et al^35^ where variation between physicians' recommendations for low‐risk PCa was identified. In their study, just under half of Urologists and oncologists did not make specific treatment recommendations, and Urologists were more likely to make recommendations in line with patients' preferences.

Compliance with long‐term monitoring of PCa, where patients fail to return for clinical review after initial consultation and agreement of a treatment path, is an ongoing concern to urology clinicians.^8^, ^31^ Embedded within clinics' patient review processes, engagement with an AS protocol may be sustained if patients consider their early interactions with the Urology clinic have addressed their needs and that their prostate health will be expertly, and proactively, monitored.

To optimize engagement with the clinic service, the information gaps^36^ and psychosocial needs^7^ indicated by patients in this study could be addressed by the introduction of a structured process to gather, utilize and disseminate information within routine clinical care, and incorporate patients' recommendations for more effective risk communication with their clinicians. As described above, clinicians in this study communicated with their patients within the clinic appointment in a way that supported shared decision making. To build on this strength, we propose the use of an information exchange process to ensure patients and clinicians receive appropriate, well‐timed information in an accessible format to assist their decision making. As the specialist Prostate Cancer Nurse already provides patients and families with health literature and links to PCa support services, this process may be best coordinated through the specialist Prostate Cancer Nurse service. To our knowledge, this study is the first to derive a structured process of information exchange for treatment of low‐risk PCa from the combined perspectives of patients and clinicians. Table 6 offers brief recommendations based on our findings. The effectiveness of such an approach requires evaluation.

**Table 6:** A process for enhancing patient risk communication

**1. Information for clinicians**
During their first clinic appointment, patients could be asked to complete a proforma (written or verbally) to gather information about their priorities, information needs and expectations of the clinic service. This information could be provided to the Urologist and specialist Prostate Cancer Nurse, to have patients concerns documented and indicate areas for discussion during the initial clinic appointment.
**2. Information for patients**
*A. About the clinic service*
Prior to the first clinic appointment, patients should be sent information about the urology clinic service. This could include details about the services provided by the clinic, including the specialist Prostate Cancer Nurse service; hours of operation and availability of the clinic staff; and contact numbers for the clinic. Additionally, some patients will have psychosocial needs that may be beyond the scope of the clinic to address. To ensure that patients' psychosocial needs and related risks are fully assessed, details of a counselling service linked to the clinic should be included in the information given to patients.
*B. About how risk is calculated and expressed in PCa clinical settings*
As some patients are clearly unaware of, or do not fully understand, clinical risk classifications (low‐, medium‐ and high‐risk), and how the risk classification applies to them, patients would benefit from receiving written information about how risk is formally assessed and how Gleason scores are derived, including diagrams that explain what low‐, medium‐ and high‐risk classification means.
*C. About PCa and treatment options*
Following the initial appointment after biopsy, or whenever it is indicated that a patient is suitable to be considered for an AS protocol, patients should be provided with information about active surveillance in PCa, and the benefits and disadvantages of AS and active treatments. Patient decision support tools exist for these reasons; however, there is a need for up‐to‐date tools that are user‐designed^38^ and specific to the Australian clinic context.^35^, ^39^
*D. About self‐management and risk minimization*
Information should be created for all urology clinics to be handed to patients upon attendance that can support patient self‐management, that is, evidence‐informed health literature to address the information gaps identified by the participants in our study, including positive health behaviours and potentially harmful behaviours to avoid.
**3. Review**
For the purpose of review, patients' initial concerns that are discussed with clinicians could be formulated into a list for patients to reflect back on before they attend follow‐up review appointments. This would allow patients to consider what had initially concerned them, follow‐up on any issues further and remove any old issues that were satisfactorily addressed. It would also enable patients to raise new concerns, or discuss changes to their life circumstances, that may have arisen over the review period.

### 4.1 Limitations

Although patient recruitment was limited to patients considered suitable for AS by the two participating Urologists, there was variation within the patient group. Not all patients had PCa, and not all were using AS. Nevertheless, this variation resulted in more complex data, deepening our understanding of how patients appraise risk and make decisions based on this appraisal. Time frame sampling was a strength in accessing a more naturalistic sample, but because of the imposed time constraint, limited the sample size.

This study has generated a hypothesis about effectiveness rather than proving effectiveness with a small participant cohort. Evaluation of the effectiveness of the approach we are suggesting has the potential to optimize engagement between health‐care professionals and patients and amongst health‐care professionals, leading to greater shared care and shared decision making within the consultation. While this is currently missing, evaluation of effectiveness would form an invaluable element of further research, using both quantitative, demonstrable measures and qualitative, in‐depth assessment to clarify the use and value of a new information exchange system for all concerned.

The study took place in a single private health clinic using a small patient and clinician cohort. Patients were from Anglo‐Australian backgrounds and well‐educated, which may reflect those patients interested in research participation. Only one Urologist participated in an interview, preventing comparison between interview the Urologists involved. Consultations with the Prostate Cancer Nurse also were unable to be observed. As such, the findings are unable to be generalized to larger or publicly funded urology services. Nevertheless, we have gained rich and extensive insight into how men from diverse age groups and life stages appraise risk and identify their priorities and information needs, which we think have scope for other urology studies.

Female researchers interviewed male patients and clinicians in a specifically male health area. It is unknown whether this had any impact on recruitment to the study or the type of information participants were willing to divulge.

## 5 CONCLUSION

Men living with localized PCa, using an AS protocol to manage their condition, appraise risk in interrelated ways. Understanding of the risks they face, and awareness of their personal priorities, formed the basis of discussions with their clinicians. Our findings that patient's appraisal of risk was influenced by their priorities and information sources indicate that effective risk communication in PCa could be enhanced by well‐timed provision of information aligned to patients' priorities, to reduce anxiety and uncertainty. Patients and clinicians could benefit from a structured process to identify patients' priorities prior to consultation, where the complexities of risk are discussed in ways that are meaningful to them. Additionally, access to a specialist Prostate Cancer Nurse contributed to the quality of, and satisfaction with, the service offered by the clinic, providing a valuable model of service delivery incorporating patient‐centric risk assessment procedures and care. By *explicitly* addressing patients' needs, patient engagement with the clinic service may be enhanced over time.

## CONFLICT OF INTEREST

The authors have no conflict of interest to declare.

## ETHICAL APPROVAL

Ethical approval for the study has been granted by Macquarie University Human Research Ethics Committee (HREC), approval number 5201600638. Knowledge translation will be achieved through publications, reports and conference presentations to patients and families, health professionals and researchers.

## DATA SHARING STATEMENT

Data available on request from the authors.
