# Supplementary material for: Appraising risk in active surveillance of localized prostate cancer
Source: Health Expect. 2019 May 16;22(5):1028–39. doi: 10.1111/hex.12912 (PMC6803412; doi:10.1111/hex.12912)
Supplement: Supplementary file 1 [file HEX-22-1028-s001.docx]

Appendices

**Appendices**

**Appendix 1 Patient Survey**

Please complete the following form by circling the relevant answers in pen. If you wish to qualify your answer, please provide a short statement after the question about your response:

1. How long ago were you diagnosed with prostate cancer?
   1. In the last few days or weeks
   2. Within the last 3 months
   3. 3-6 months ago
   4. 6-12 months ago
   5. 1-2 years ago
   6. Over 2 years ago

______________________________________________________________________________________________________________________________________

1. Apart from the Macquarie University Hospital and Clinic, please list all other medical services where you have received treatment for cancer.

___________________________________________________________________

______________________________________________________________________________________________________________________________________

1. Approximately how many times have you been to see a doctor or other health professional about your prostate cancer?
   1. 1-2
   2. 2-5
   3. 5-10
   4. Greater than 10

______________________________________________________________________________________________________________________________________

1. Have you made any definitive decisions regarding treatment for your prostate cancer?
   1. No
   2. Yes - If yes, please list what treatment you have decided upon and why

_________________________________________________________________________________________________________________________________________________________________________________________________________

1. Do you know your current risk classification for prostate cancer?
   1. No
   2. Yes - If yes, please state your risk classification

______________________________________________________________________________________________________________________________________

1. What sources of information have you used to get details of the risks that are involved with your prostate cancer? (Please check all that apply)
   - Doctors in the clinic
   - Doctors working in the hospital
   - Nurses and other health professionals
   - Patient information booklets
   - The internet
   - Friends/family members
   - Other, please specify

_________________________________________________________________________________________________________________________________________________________________________________________________________

1. What are your priorities regarding the management of your prostate cancer? Please rank items from 1-6, 1 being the most important, and 6 being the least important to you.
   - Staying physically healthy and living a long life
   - Emotional/mental/social wellbeing
   - Retaining good physical functioning
   - Limiting the impact of prostate cancer on your life
   - Limiting the impact of prostate cancer treatment on your life
   - Having good-quality relationships with the people in your life
2. What age range do you fall into?
   1. 18-39
   2. 40-49
   3. 50-64
   4. 65-79
   5. 80+
3. What is your sexual orientation?
   1. Heterosexual/straight
   2. Homosexual/gay
   3. Bisexual
   4. Other – I identify as… (Please specify)

______________________________________________________________________________________________________________________________________

1. What is your relationship status?
   1. Single
   2. Married/de facto
   3. Separated/divorced
   4. Widowed

______________________________________________________________________________________________________________________________________

1. What is your cultural background?
   1. Anglo-Australian
   2. European
   3. Aboriginal/Torres Strait Islander
   4. Asian
   5. Middle eastern
   6. African
   7. Other – I identify as… (Please specify)

______________________________________________________________________________________________________________________________________

1. What is your highest level of education attained?
   1. High school
   2. Trade/apprenticeship qualification
   3. Certificate/diploma
   4. University degree or higher
   5. Other – (Please specify)

______________________________________________________________________________________________________________________________________

**Appendix 2 Observation checklist**

A member of the study team will observe consultations and take note of the following:

1. Discussions of treatment options
2. The discussion or use of any risk assessment procedures (e.g. Gleason score, prostate specific antigen testing, MRI, biopsy)
3. Whether, how and by whom (patient or clinician) risk is brought up
4. Why risk brought up
5. The language used to convey risk (e.g. probabilities, jargon, or concerns surrounding risks
6. Relationship between risk as a clinical and risk as a non-clinical concern (e.g. social, emotional)
7. Differences and similarities in patient and clinician understanding and expression of risk
8. How decisions are made around risk management and treatments, and whether they are negotiated
9. The way that negotiation is expressed and concluded.
10. Emotive expressions of risk during consultations.
11. Body language and other non-verbal exchanges of patients and clinicians during discussions related to risk, prostate cancer and active surveillance

**Appendix 3 Patient interview schedule**

The patient interview will cover the following topics:

1. Experiences of information about risk in the oncology consultation
   1. Example: Can you tell me a bit about your experience of being diagnosed with prostate cancer? How did you come to understand what that meant?
2. Personal definitions of risk in the context of prostate cancer and active surveillance
   1. Example: What do you consider to be a risk in prostate cancer? Why?
3. Their priorities with regard to risks in prostate cancer and active surveillance
   1. Example: What are you most concerned about with your diagnosis of prostate cancer?
4. Whether priorities regarding risk in prostate cancer and active surveillance have changed.
   1. Example: Have your concerns changed since you were initially diagnosed?
5. The extent they consider these priorities as taken into account by health professionals
   1. Example: How have your doctor and other health professionals responded to your concerns?
6. Views and experiences on risk assessment and their risk classification
   1. Example: Have you undergone a formal risk assessment for your prostate cancer? If so, what was that like? What is your risk classification?
   2. Example: If not, has your doctor provided you details regarding your risk in prostate cancer? Have they talked about prognosis?
   3. Example: Can you tell me about your experience of prostate cancer testings? How have you found receiving the results of these tests?
7. Perceptions and expectations of active surveillance
   1. Example: What do you know about active surveillance?
8. How active surveillance has been discussed with health professionals
   1. Example: What has your doctor told you about active surveillance? How useful has this information been?
9. Treatment decisions, how they are made and by whom, how care is negotiated with healthcare professionals.
   1. Example: What about discussing other treatments with your doctor?
10. Their experiences of discussing risk in clinical consultations for prostate cancer
    1. Example: Can you tell me about your experience of talking to your doctors about your diagnosis and what it means?
11. Patients’ aspirations for future health and well-being
    1. Example: What are your hopes for your treatment?

**Appendix 4 Clinician interview schedule**

The clinician interview will cover:

1. Personal definitions of risk in the context of prostate cancer and active surveillance
   1. Example: What do you consider to be a risk in prostate cancer? Why?
2. Experiences of information about risk in the oncology consultation
   1. Example: Can you tell me a bit about your experience of explaining these risks to patients who have prostate cancer?
3. Their priorities with regard to risks in prostate cancer and active surveillance
   1. Example: What are you most concerned about when a patient is diagnosed with localised prostate cancer? Is this different for different patients?
4. How they understand patients’ needs for communication about risk in prostate cancer and active surveillance.
   1. Example: What do you consider to be patients’ biggest concerns following a diagnosis of prostate cancer?
5. The extent they take into account patients’ priorities
   1. Example: How do you respond to these concerns?
6. Views and experiences on risk assessment and their risk classification
   1. Example: How have you found communicating the results of these tests with patients?
7. Perceptions and expectations of active surveillance
   1. Example: What are your views on active surveillance? Do you discuss active surveillance with your patients? How? What sort of patients?
8. Treatment decisions, how they are made and by whom, how care is negotiated with patients.
   1. Example: How does your patients usually come to a definitive decision regarding active surveillance or other treatments? What role do you try to play in that decision?
9. Benefits and disadvantages of risk communication/ formal risk assessment
   1. Example: What do you see as the biggest issues with communicating with patients diagnosed with localised prostate cancer about risk?
10. Impact of risk communication on shared decision-making around treatment
    1. Example: How do you find your discussions about patients’ risks and prognosis affect their treatment decisions? How does it affect the way those decisions are made?
